# Current complementary and alternative therapy forgastroesophageal reflux disease

**DOI:** 10.1093/gastro/goad057

**Published:** 2023-10-04

**Authors:** Dianxuan Jiang, Qianjun Zhuang, Xingyu Jia, Songfeng Chen, Niandi Tan, Mengyu Zhang, Yinglian Xiao

**Affiliations:** Department of Gastroenterology, The First Affiliated Hospital of Sun Yat-sen University, Guangzhou, Guangdong, P. R. China; Department of Gastroenterology, The First Affiliated Hospital of Sun Yat-sen University, Guangzhou, Guangdong, P. R. China; Department of Gastroenterology, The First Affiliated Hospital of Sun Yat-sen University, Guangzhou, Guangdong, P. R. China; Department of Gastroenterology, The First Affiliated Hospital of Sun Yat-sen University, Guangzhou, Guangdong, P. R. China; Department of Gastroenterology, The First Affiliated Hospital of Sun Yat-sen University, Guangzhou, Guangdong, P. R. China; Department of Gastroenterology, The First Affiliated Hospital of Sun Yat-sen University, Guangzhou, Guangdong, P. R. China; Department of Gastroenterology, The First Affiliated Hospital of Sun Yat-sen University, Guangzhou, Guangdong, P. R. China

**Keywords:** GERD, complementary therapies, alternative therapies, lifestyle, traditional Chinese medicine, behavior intervention

## Abstract

Gastroesophageal reflux disease (GERD) is a widely prevalent gastrointestinal disorder, affecting ∼13.3% of the global population. There are shortages and limitations of current GERD treatment modalities, and complementary and alternative therapy (CAT) is a promising option to fill in the gap. Dietary and lifestyle modifications might play an important and complementary role in alleviating GERD symptoms. Traditional Chinese medicine and brain–gut behavior therapy, particularly transcutaneous electrical acustimulation and diaphragmatic breathing therapy were shown to be useful adjuncts or alternatives in treating GERD. CAT may help to relieve GERD symptoms, minimize medication dosage, and slow the demand for surgery. The aim of this review was to summarize the existing evidence of some common CATs in treating symptomatic GERD, including dietary modification, lifestyle change, traditional Chinese medicine, and brain–gut behavior therapy.

## Introduction

Gastroesophageal reflux disease (GERD) is a common chronic gastrointestinal disease that affects ∼13.3% of the population worldwide [1]. Owing to its high prevalence and chronic nature, GERD seriously affects patients’ quality of life and places a substantialeconomic burden on society [2, 3]. The annual costs related to GERD have reached $10 billion in the USA [4].

Conventional treatment modalities for GERD include pharmacologic therapy, and surgical and endoscopic interventions. Proton-pump inhibitors (PPIs) are the first-line treatment for GERD. However, up to 50% of patients reported persisting reflux symptoms despite PPI use [5]. In addition, long-term PPI use was associated with some adverse events, including bone fractures, kidney disease, infections, and gastric cancer [6]. Patients whoare refractory to medications or refuse long-term PPI use may undergo surgical or endoscopic interventions. At present, laparoscopic anti-reflux surgery (LARS) is the mainstay procedure in surgical options. However, a cohort study including 2,655 patients who underwent LARS reported that 17.7% of patients needed medications or secondary surgery after primary LARS, and 4% of patients suffered from post-operative complications [7]. Besides, the long-term efficacy of some emerging endoscopic procedures remains controversial [8, 9].

As limitations exist in pharmacologic and surgical treatment, there is growing interest in applying complementary and alternative therapy (CAT) to optimize GERD management. Dietary and lifestyle modifications can add as effective complements for GERD management [10]. Clinical guidelines suggested that avoidance of trigger foods and late meals, tobacco cessation, weight loss, and elevation of the bed head during sleep were beneficial for symptomatic GERD patients [11, 12]. However, the benefits of dietary pattern changes and physical exercise in GERD management are controversial. Besides, recent studies have shown traditional Chinese medicine (TCM) and brain–gut behavior therapy (BGBT) are effective in treating GERD, including transcutaneous electrical acustimulation (TEA) and diaphragmatic breathing therapy (DB), which may be promising adjuncts or alternatives for patients who fail medical and surgical therapies. This review summarizes the evidence of some common CATs in GERD management,including dietary and lifestyle modification, TCM, and BGBT.

## Dietary modification

Several studies have indicated that unhealthy dietary structure or eating habits may be risk factors for GERD. Changes to dietary composition, eating habits, and diet patterns may lead to significant improvement in GERD symptoms (Table 1). In a clinical trialof 100 GERD patients, almost half of the patients reported symptom relief and PPI cessation after 2 weeks of dietary modification [13].

**Table 1.** Prospective studies on dietary modification for symptomatic GERD

Author	Study design	Subject	Intervention	Follow-up duration	Outcome
Pehl *etal.* (1999) [16]	Non-RCT	12 healthy volunteers	Low-fat vs high-fat	–	No difference in the frequency of TLESRs, AET, and LESP was observed
Penagini *etal.* (1998) [15]	Non-RCT	13 healthy volunteers and 14 GERD patients	High-fat vs balanced fat	–	No difference in esophageal acid exposure, the rate of reflux episodes, the rate of TLESRs, and basal LESP was observed
Fox *etal.* (2007) [17]	Non-RCT	15 GERD patients	High-calorie vs low-calorie	–	High-calorie diet led to AET increase*
Pointer *etal.* (2016) [19]	Non-RCT	144 obese women	High-fat/low-carbohydrate diet	16 weeks	Total carbohydrates, total sugars, GERD symptoms, and medication usage decreased
Gu *etal.* (2022) [20]	RCT	98 GERD patients	HTHS (*n *=* *22) vs HTLS (*n *=* *26) vs LTHS (*n *=* *22) vs LTLS (*n *=* *25)	9 weeks	Reduction in simple sugar intake led to decrease in AET* and reflux episodes* and overall improvement of symptoms
Morozov *etal.* (2018) [22]	Non-RCT	36 non-erosive gastroesophageal reflux disease patients	A fiber-enriched diet	10 days	The frequency of heartburn***, the number of refluxes***, and minimal resting LESP decreased*
Aanen *etal.* (2006) [23]	Crossover RCT	10 healthy volunteers	5 g of NaCl vs placebo in capsules per day	1 week	No difference in the number of reflux episodes and TLESRs was observed
Tosetti *etal.* (2021) [13]	Non-RCT	100 GERD patients	Elimination of specific food items	2 weeks	Overall improvement of symptoms was observed
Mehta *etal.* (2020) [29]	Cohort study	48,308 women	–	262,641 person-years	Coffee, tea, and soda consumption were risk factors for GERD
Patcharatrakul *etal.* (2021) [33]	Crossover RCT	21 patients with overlapped GERD and irritable bowel syndrome	Wheat noodles vs rice noodles	–	Wheat ingestion increased heartburn and regurgitation scores*
Rivière *etal.* (2021) [32]	RCT	31 refractory GERD patients	A low-FODMAP diet (*n *=* *16) vs usual diet (*n *=* *15)	4 weeks	No difference in total Reflux Disease Questionnaire score, acid exposure, and reflux episodes was observed
Wu *etal.* (2014) [37]	Non-RCT	15 GERD patients	High-volume vs low-volume	2 consecutive days	A high-volume meal increased total number of reflux episodes*, AET*, and the number of reflux symptoms**
Wildi *etal.* (2004) [38]	Non-RCT	20 healthy volunteers	Fast vs slow eating	2 consecutive days	Rapid food intake increased the number of reflux episodes*
Bor *etal.* (2013) [39]	Non-RCT	46 GERD patients	Fast vs slow eating	2 consecutive days	No difference in the total number of refluxepisodes was observed
Valitova *etal.* (2013) [40]	Non-RCT	60 GERD patients	Fast vs slow eating	2 consecutive days	No difference in the number of reflux episodes and symptoms was observed
Piesman *etal.* (2007) [42]	Crossover RCT	32 GERD patients	Early vs late meal	2 consecutive days	An early meal decreased supine AET** and the number of nocturnal episodes*

AET, acid exposure time; FODMAP, fermentable, oligosaccharides, disaccharides, monosaccharides, and polyols; GERD, gastroesophageal reflux disease; HTHS, high total/high simple carbohydrate; HTLS, high total/low simple carbohydrate; LESP, lower esophageal sphincter pressure; LTHS, low total/high simple carbohydrate; LTLS, low total/low simple carbohydrate; RCT, randomized–controlled trial; TLESRs, transient lower esophageal sphincter relaxations.

* *P *<* *0.05;

** *P *≤* *0.01;

*** *P *≤* *0.001.

### Modification of dietary composition

Different nutrients have various impacts on GERD symptoms. A high-fat diet was considered as a trigger for GERD symptoms in observational studies [14]. However, studies showed that the percentage of postprandial reflux, frequency of transient lower esophageal sphincter relaxations (TLESRs), and acid exposure time (AET) were similar after a low-fat and high-fat meal among healthy volunteers and GERD patients [15, 16]. Therefore, instead of fat content, caloric density is assumed to be blamed for reflux. A study by Fox *etal.* [17] showed that patients exhibited more acid exposure on the high-calorie diet than the low-calorie one, regardless of fat content.

Although no correlation between total carbohydrate intake and GERD was found in population-based studies, a crossover placebo-controlled study showed that a high-carbohydrate diet might enhance colonic fermentation and worsen reflux symptoms [14, 18]. Besides, an interventional trial indicated that a low-carbohydrate diet was associated with the resolution of GERD symptoms and reduced medication use [19]. A randomized–controlled trial (RCT) including 98 symptomatic GERD patients also demonstrated that reduced intake of simple sugar could lead to a decrease in the AET, frequency, and severity of symptoms [20].

High dietary fiber intake can reduce the risk of GERD. Two large population-based studies have shown an inverse relationship between fiber intake and the risk of GERD [14, 21]. Morozov *et al*. [22] further showed a reduction in the frequency of heartburn and the number of reflux episodes after a 10-day fiber-enriched diet. On the contrary, high salt intake may be a risk factor for GERD. A dose-dependent relationship between higher salt intake and an increased risk of GERD was observed, but a placebo-controlled trial showed no impacts of high salt intake on the number of reflux episodes or TLESRs [21, 23]. The effects of high salt intake on GERD need further investigation.

Furthermore, some specific foods have been reported as risk factors for GERD, including meat (odds ratio [OR] 1.09, 95% confidenceinterval [CI] 1.04–1.14), fried foods (OR 3.01, 95% CI 1.52–6.20), spicy foods (OR 1.09, 95% CI 1.02–1.16), citrus fruit (OR 2.22, 95% CI 1.3–3.81), and sweets (OR 1.42, 95% CI 1.00–2.02), while some foods were protective factors, such as vegetables (OR 0.34, 95% CI 0.21–0.54) and vitamins (OR 0.46, 95% CI 0.24–0.90) [24, 25]. Restriction of trigger foods was beneficial to ameliorate symptoms [13]. However, data on the relationship between specific foods and GERD are inconclusive, further studies with larger sample sizes and long-term follow-up are needed.

As for beverages, several systematic studies have found no correlations between alcohol, coffee, tea, and soft drinks and GERD; however, most of the included studies were retrospective or cross-sectional [26–28]. Challenging these results, a prospective cohort study comprising 48,308 females suggested dose-dependent associations between the consumption of coffee, tea, and carbonated beverages and an increased risk of GERD [29]. A large population-based study conducted in Japan involving 19,864 adults also found a positive association between alcohol consumption and GERD symptoms [30]. The American College of Gastroenterology guideline on GERD management recommended that GERD patients should avoid trigger beverages [12], but the definitive associations between specific beverages and GERD remained to be explored.

In summary, avoidance of trigger food, including specific nutrients, foods, and beverages, is recommended in GERD management. A low-calorie, low-carbohydrate, fiber-enriched diet may also be recommended for GERD. However, the definition and the margin of “low-calorie, low-carbohydrate and fiber-enriched” remain unknown.

### Adherence to specific dietary patterns

GERD has gradually become a global problem with the popularization of Western dietary patterns [31]. Switching dietary pattern to a low-FODMAP or Mediterranean diet may be beneficial for symptom relief. A low-FODMAP diet is characterized by low fermentable, oligosaccharides, disaccharides, monosaccharides, and polyols [32]. A crossover RCT in 21 patients with overlapped GERD and irritable bowel syndrome demonstrated that a low-FODMAP diet significantly reduced postprandial GERD symptoms and the number of TLESRs [33]. However, Rivière *etal.* [32] found no significant difference between a 4-week low-FODMAP diet and the usual diet in relieving PPI-refractory GERD symptoms. The Mediterranean diet, characterized by plant-based food and low contents of saturated fat, has been reported as beneficial to health status [34]. In a cross-sectional study conducted in Albania, a Mediterranean diet was correlated with a decreased risk of GERD, irrespective of lifestyle factors and dietary habits [35].

### Modification of dietary habits

In addition to dietary components and patterns, dietary habits in terms of the volume, speed, and timing of meals may also contribute to GERD symptoms. A high-volume meal might increase TLESRs and induce reflux [36]. A small controlled trial including 15 GERD patients showed that a high-volume meal significantly increased AET, the total number of reflux episodes, and symptoms as compared with a low-volume meal [37]. Rapid food intake was assumed to increase reflux as well [38]. However, two controlled trials showed no difference between fast- and slow-eating groups regarding either the number of reflux episodes or the severity of symptoms [39, 40]. Shorter dinner-to-bed time has been identified as a risk factor for GERD [41]. A crossover RCT further assessed its effects on GERD [42]. There were longer supine AET and more nocturnal reflux episodes in the late-meal group (2 h before going to bed) as compared with the early-meal group (6 h before going to bed).

On the basis of the available data, adherence to a low-FODMAP diet is recommended in patients with overlapped GERD–irritable bowel syndrome, while avoidance of a high-volume or late meal has shown benefits in patients with GERD symptoms. Further studies are needed to investigate the role of the Mediterranean diet in GERD and determine the definition of a high-volume meal.

## Lifestyle modification

Several lifestyle factors have been identified as risk factors for GERD, including obesity and smoking. Besides, studies have shown the benefits of weight loss, tobacco cessation, sleeping with the head of the bed elevated, and proper exercise on GERD (Table 2). A prospective trial performed by Mehta *etal.* [43] showed a positive outcome with alterations in external lifestyle factors in GERD patients, with a 40% symptom prevention rate.

**Table 2.** Prospective studies on lifestyle change for symptomatic GERD

Author	Study design	Subject	Intervention	Follow-up duration	Outcome
Ness-Jensen *etal.* (2013) [46]	Cohort study	29,610 GERD patients	–	–	Weight loss was dose-dependently associated with a reduction in GERD symptoms
Singh *etal.* (2013) [47]	Non-RCT	332 overweight/obese subjects	Weight loss program	6 months	Weight loss led to a decrease in waist circumference, body mass index, and GERD symptom scores
Ness-Jensen *etal.* (2013) [50]	Cohort study	29,610 GERD patients	–	–	Tobacco cessation was associated with improvement in symptoms
Kohata *etal.* (2016) [51]	Non-RCT	191 subjects	Smoking cessation	1 year	A reduction in the prevalence of GERD* and overall improvement of symptoms was observed
Khan *etal.* (2012) [53]	Non-RCT	24 patients with nocturnalreflux	Sleeping with head elevated by 20 cm	7 days	Supine reflux time*** and symptom scores decreased**
Person *etal.* (2015) [55]	Crossover RCT	20 healthy volunteers	Lying right-side down vs lying left-side down vs lying on a wedge vs lying flat	4 nights	Lying left-side down led to the lowestacid exposure
Schuitenmaker *etal.* (2022) [56]	RCT	100 patients with nocturnalGERD	Sleeping left-side down (*n *=* *50) vs sham-controlled(*n *=* *50)	2 weeks	Lying left-side down reduced nocturnalreflux symptoms
Mehta *etal.* (2021) [43]	Cohort study	42,955 women	–	392,215 person-years	Adherence to moderate-to-vigorousactivity was associated with a decreased risk of GERD

GERD, gastroesophageal reflux disease; RCT, randomized–controlled trial.

* *P *<* *0.05;

** *P *≤* *0.01;

*** *P *≤* *0.001.

### Weight loss

Obesity is associated with an increased risk of GERD. It was reported that the prevalence of GERD symptoms was 22% in overweight subjects [1]. The underlying mechanisms include elevated intra-abdominal and intra-gastric pressures, esophageal motility disorders, impaired esophageal mucosal barrier, and increased frequency of TLESRs [28, 44]. In a large population-based study of 10,545 women from the Nurses' Health Study, a dose-dependent association between increasing body mass index and severity of GERD symptoms was observed in both normal-weight and overweight women [45]. A genetic study further found a casual role of high body mass index in the development of GERD (OR 1.49, 95% CI 1.40–1.60) [28].

Weight loss was proven to be beneficial to prevent and treat GERD. Ness-Jensen *etal.* [46] prospectively enrolled 29,610 subjects and found that weight loss was not only associated with a reduction in symptoms, but also improved the treatment outcome of medical therapy. In an interventional trial, 332 overweight/obese subjects who received a structured weight loss program exhibited a significant decrease in the prevalence of GERD and symptom scores after 6 months [47]. The percentage of weight loss was positively correlated with symptom improvement (*P *<* *0.05).

### Tobacco cessation

Smoking worsens reflux symptoms. Tobacco consumption was shown to increase the risk of GERD by 1.12- to 1.70-fold in several population-based studies [21, 30, 48, 49], while smoking cessation could benefit GERD patients. In a cohort study of 29,610 subjects, tobacco cessation was associated with an improvement in GERD symptoms [50]. However, this association was only present among individuals using medication at least weekly or within the normal range of body mass index. Besides, the long-term effects of smoking cessation were confirmed by Kohata *etal.* [51]. Patients who quit smoking for a year had significant improvements in GERD symptoms and overall quality of life as compared with those who did not.

### Bed head elevation during sleep

A reduction in nocturnal reflux was associated with a high quality of sleep [52]. In a clinical trial, patients with supine reflux had a significant reduction in supine AET and symptom score after sleeping with head elevated by 20 cm for 7 days [53].

In addition, the sleep position also plays a role. There was evidence that lying right-side down increased the incidence of TLESRs and induced more reflux episodes [54], while two RCTs demonstrated the superiority of lying left-side down [55, 56]. Lying left-side down was significantly associated with lower AET and decreased reflux episodes among healthy subjects, and a sham-controlled study including 100 patients with nocturnal GERD showed that avoidance of right lateral decubitus significantly reduced nocturnal symptoms and improved the sleep quality.

### Proper physical exercise

Physical exercise seems to be a double-edged sword for GERD. Strenuous exercise may exacerbate GERD while moderate recreational exercise may have the opposite effect. Vigorous exercise could induce reflux and greater acid exposure in both healthy volunteers and athletes [57]. However, some cross-sectional studies have demonstrated that less physical exercise was a risk factor for GERD (the range of OR: 6.47–7.03), while moderate- or high-intensity physical exercise was associated with a decreased risk of GERD (the range of OR: 0.32–0.83) [30, 58, 59]. Moreover, prospective data showed that adherence to moderate-to-vigorous physical activity of ≥30 min a week was correlated with a decreased risk of GERD (the range of OR: 0.4– 0.9) [21, 43].

Taken together, lifestyle change, including weight loss, tobacco cessation, and sleeping with bed head elevation, is generally beneficial for symptomatic GERD patients. Although observational studies have found the association of proper exercise with a decreased risk of GERD, the degree of “proper” remains unclear. High-quality evidence on the effects of exercise on GERD patients is insufficient.

## TCM

### Herbal medicine

Herbal medicine, especially traditional Chinese herbal medicine, has long been used to treat GERD. As potential alternative options for GERD management, several TCM formulas have been shown non-inferior to Western medications in terms of symptom relief in RCTs, including JianpiQinghua granule, Wu chu yu tang, Tongjiang Granule, Modified Xiaochaihu Decoction, and Hewei Jiangni Decoction (Table 3) [60–64]. Among them, JianpiQinghua granule and Tongjiang Granule were superior to Western medications in symptom control. Meta-analyses also demonstrated that Wendan Decoction, Sini Zuojin Decoction, and Modified Banxia Xiexin Decoction had greater clinical efficacy as compared with Western medications [65–67]. From the prospective of TCM, the pathogenesis of GERD includes liver depression and spleen deficiency, and disharmony between the liver and stomach. Most of the formulas play a role in invigorating the spleen, regulating the liver, and harmonizing the stomach [68]. *Evodiae fructus* (Wu-Chu-Yu) and Coptidis Rhizoma (Huanglian) are the core ingredients of these formulas, which possibly target the anti-inflammatory pathway [69]. Although herbal formulas alone or in combination with Western medications have achieved certain efficacy in the treatment of GERD, their key components and protective mechanisms remain to be elucidated.

**Table 3.** Prospective studies on traditional Chinese medicine for symptomatic GERD

Author	Study design	Subject	Intervention	Follow-up duration	Outcome
Zhang *etal.* (2021) [63]	RCT	204 GERD patients	Jianpi Qinghua granule + half-dose omeprazole (*n *=* *98) vs omeprazole (*n *=* *98)	4 weeks	Jianpi Qinghua granule increased complete resolution rate*
Shih *etal.* (2019) [61]	RCT	90 GERD patients	Wu chu yu tang (*n *=* *40) vs omeprazole (*n *=* *37)	4 weeks	No difference in Reflux Disease Questionnaire and GERDQ was observed
Li *etal.* (2011) [60]	RCT	120 non-erosive reflux diseasepatients	Tongjiang Granule (*n *=* *57) vs mosapride citrate (*n *=* *55)	4 weeks	Tongjiang Granule increased effective rate**
Li *etal.* (2021) [62]	RCT	288 GERD patients	Modified Xiaochaihu decoction (*n *=* *39) vs omeprazole (*n *=* *41)	4 weeks	Similar symptom control (GERDQ) but improvement of esophageal motility (LESP, the percentage of IEM) on modifiedXiaochaihu decoction was observed
Li *etal.* (2022) [64]	RCT	128 non-erosive reflux diseasepatients	Hewei Jiangni Decoction (*n *=* *56) vs omeprazole (*n *=* *53)	8 weeks	Similar efficacy (GERDQ and patient-reported outcomes) was observed
Meng *etal.* (2016) [73]	RCT	20 refractory GERD patients	ESO vs ESO + TEA vs ESO + sham TEA vs ESO + domperidone (*n *=* *5, each group)	4 weeks	A significant increase in LESP and reductionin weak acid reflux was only observed in ESO + TEA group
Hu *etal.* (2020) [74]	RCT	30 GERD patients	TEA (*n *=* *15) vs sham TEA (*n *=* *15)	30 min	Overall improvement of gastrointestinal symptoms (postprandial fullness and belching) on TEA was observed
Zhang *etal.* (2021) [75]	RCT	30 GERD patients with IEM	TEA (*n *=* *15) vs sham TEA (*n *=* *15)	4 weeks	Overall improvement of gastrointestinal symptoms (postprandial fullness and belching) and esophageal motility (LESP and distal contractile integral) on TEA was observed
Liu *etal.* (2019) [76]	RCT	60 GERD patients	STEA (*n *=* *45) vs sham TEA (*n *=* *15)	30 min	Improvement of esophageal motility (LESP and the percentage of IEM) and vagal activity (HF, LF, and LF/HF) on STEA was observed
Yu *etal.* (2019) [77]	RCT	21 refractory GERD patients	TEA + diaphragmatic breathing therapy + ESO vs sham TEA + diaphragmaticbreathing therapy + ESO vs ESO (*n *=* *7, each group)	4 weeks	Improvement in reflux symptoms, LESP, and vagal activity (LF/LF + HF; HF/LF + HF) on TEA + diaphragmatic breathingtherapy + ESO was observed

ESO, esomeprazole; GERD, gastroesophageal reflux disease; GERDQ, GERD questionnaire; HF, high-frequency; IEM, ineffective esophageal motility; LESP, lower esophageal sphincter pressure; LF, low-frequency; RCT, randomized–controlled trial; STEA, transcutaneous electrical acustimulation in synchronization with inspiration; TEA, transcutaneous electrical acustimulation; +, plus.

* *P *<* *0.05;

** *P *≤* *0.01.

### Acupuncture

As a part of TCM, manual acupuncture and electroacupuncture have been reported effective in various gastrointestinal disorders, including GERD [70]. The major acupoints are ST36 (Zusanli) and PC6 (Neiguan). A meta-analysis showed that manual acupuncture/electroacupuncture plus Western medications led to significant symptom improvement, while manual acupuncture/electroacupuncture alone was associated with a lower recurrence rate as compared with Western medications [71]. However, manual acupuncture/electroacupuncture requires frequent visits to hospital and is performed under the supervision of professionals, which limits its wide use.

Recently, TEA, a non-invasive and needleless acupoint stimulation approach, has been applied for the treatment of GERD [72]. It can be performed by patients at home daily or even a few times daily. Meng *etal.* [73] concluded that those who received PPI plus TEA for 4 weeks had significantly higher lower esophageal sphincter pressure and symptom improvement than patients treated with PPI alone or PPI plus sham TEA. The potential mechanisms of TEA on GERD might be to enhance vagal activity, improve gastric accommodation, restore esophageal body motility, and normalize impaired gastric slow waves [74, 75].

TEA in synchronization with inspiration (STEA) was also shown to enhance vagal activity. During STEA, each electrical stimulus was in synchronization with inspiration. Similar to TEA, STEA had a significant impact on enhancing esophageal motility and relieving reflux symptoms in GERD patients [76, 77]. Though short-term benefits of TEA or STEA were proven in RCTs, further studies should focus on their long-term effects.

There are some limitations in studies of TCM. Most of the enrolled subjects were Chinese and the efficacy of TCM in other populations was unknown. In addition, the follow-up duration and sample size were inadequate in these studies. The mechanisms of herbal medicine are also unclear.

## Brain–gut behavior therapy

GERD is associated with psychological distress, particularly anxiety and depression [78]. Poor mental state may result in reduced quality of life and treatment failure in GERD patients. Advances in behavior intervention science, especially BGBT, have enabled the improvement of these psychological co-morbidities. It targets specific cognitive, emotional, and behavioral factors and improves gastrointestinal symptoms [79]. Although the exact relationship between psychological distress and GERD is not well understood, some emerging BGBTs have shown benefits in GERD (Table 4).

**Table 4.** Prospective studies on brain–gut behavior therapies for symptomatic GERD

Author	Study design	Subject	Intervention	Follow-up duration	Outcome
Glasinovic *etal.* (2018) [81]	Non-RCT	51 patients with pathological supragastric belching	Cognitive behavioral therapy	8 weeks	The number of supragastric belching episodes***, the number of acid reflux episodes^***^, and acid exposure time** decreased
Eherer *etal.* (2012) [84]	RCT	19 GERD patients	DB + PPI (*n *=* *10) vs PPI (*n *=* *9)	4 weeks	There was a significant decrease in acid exposure time and improvement in quality of life on DB + PPI but not PPI alone
Sun *etal.* (2016) [82]	RCT	40 GERD patients	DB + rabeprazole (*n *=* *20) vs rabeprazole (*n *=* *20)	8 weeks	DB + rabeprazole significantly enhanced anti-reflux barrier (crural diaphragm tension and gastroesophageal junction pressure) compared with rabeprazole alone
Halland *etal.* (2021) [83]	RCT	23 patients and 10 healthy volunteers	DB (*n *=* *11 patients + 5 healthy volunteers) vs sham DB (remaining subjects)	2 days	DB significantly decreased lower esophageal sphincter pressure***, the number of reflux episodes***,and postprandial acid exposure*
Chandran *etal.* (2019) [88]	Non-RCT	120 GERD patients	Mindfulness-based stress reduction + PPI + lifestyle modification vs PPI + lifestyle modification	8 weeks	Intervention with mindfulness-based stress reduction led to overall improvement of symptoms (GERD-Health-Related Quality of Life and symptom scores)

DB, diaphragmatic breathing therapy; GERD, gastroesophageal reflux disease; PPI, proton-pump inhibitor; RCT, randomized–controlled trial; +, plus.

* *P *<* *0.05;

** *P *≤* *0.01;

*** *P *≤* *0.001.

Cognitive behavioral therapy, one of the most well-tested BGBTs, has been used successfully in treating a range of digestive disorders. Patients who receive cognitive behavioral therapy learn ways to control their thoughts, behaviors, and physiologic responses, thus improving their physical and mental discomfort [79]. The effects of cognitive behavioral therapy have been investigated on excessive supragastric belching, which is a behavior disorder highly associated with GERD [80]. In an interventional trial of 39 patients with pathological supragastric belching, 8-week cognitive behavioral therapy significantly decreased the number of supragastric belching and acid reflux episodes, as well as AET [81].

Another promising BGBT is DB. Similar to TEA, DB helps to increase vagal activity and improve gastric and esophageal motility. Studies also showed that either acute (30 min) or chronic (8 weeks) DB could significantly enhance the function of the anti-reflux barrier [82, 83]. Furthermore, another RCT found a significant reduction in AET and GERD symptoms after 4 weeks of DB [84]. After 9 months of training, medication use was significantly reduced in patients.

Mindfulness-based therapy is rooted in being calm at the present moment via meditation and relaxation [79]. On mindfulness-based therapy, patients can reduce suffering pain and improve their moods [85]. There was evidence that patients with GERD exhibited higher scores of depression and anxiety, and impaired quality of life [86, 87]. Patients with GERD showed significant improvements in anxiety, depression, overall GERD symptoms, and quality of life after 8 weeks of mindfulness-based therapy [88].

Although there is strong evidence to support the application of BGBTs in a wide range of digestive disorders, the efficacy data on GERD are scarce. In addition to DB, cognitive behavioral therapy and mindfulness-based therapy hold promise for being useful adjuncts or alternatives in treating GERD.

## Conclusions and recommendations

In the past decades, acid-suppressive therapy and surgical interventions have been the mainstay treatments for GERD, but they are followed by unsatisfying outcomes and adverse events. CAT may play a complementary or alternative role in treating GERD. In this review, we have concluded some evidence of CAT on GERD management.

In terms of dietary modification, avoidance of trigger foods or late meals is a complementary option for patients with GERD symptoms, while adherence to a low-FODMAP diet has shown benefits in patients with overlapped GERD–irritable bowel syndrome. In terms of lifestyle change, weight loss, smoking cessation, and sleeping with bed head elevation are beneficial for symptom control. As for potential alternatives, treatment outcomes of TCM and BGBTs are promising. However, high-quality RCTs with long-term follow-up and large sample sizes are needed.

In conclusion, complementary therapies, including aviodance of trigger foods or late meals, adherence to a low-FODMAP diet, weight loss, smoking cessation, and sleeping with bed head elevation, are recommended for GERD management, while the efficacy of alternative treatments, including TCM and BGBT, remains to be validated in the future.

## Authors’ Contributions

D.J. and Q.Z. reviewed the literature, collected data, and drafted the manuscript; Y.X. conceived and designed the study and finalized the manuscript; X.J., S.C., N.T., and M.Z. revised the manuscript. All authors have read and approved the final version of the manuscript.
